# Development of Acquired Factor V Inhibitor After Surgical Procedure Without the Use of Fibrin Tissue Adhesives: A Case Report

**DOI:** 10.7759/cureus.12708

**Published:** 2021-01-14

**Authors:** Hirohisa Hirata, Yoshihiko Sakurai, Tomohiro Takeda, Tetsuya Kasetani, Takeshi Morita

**Affiliations:** 1 Department of Surgery, Matsubara Tokushukai Hospital, Matsubara, JPN; 2 Department of Pediatrics, Matsubara Tokushukai Hospital, Matsubara, JPN; 3 Department of Clinical Laboratory Science, Kansai University of Health Sciences, Kumatori-cho, JPN

**Keywords:** factor v, inhibitor, bovine thrombin, cross-mixing test, heteroantibody, autoantibody, tissue adhesive

## Abstract

Development of acquired factor V (FV) inhibitor is a rare coagulation disorder. Production of heteroantibodies against bovine FV, a contaminant in fibrin tissue adhesives, is a common cause of this condition in the field of surgery. The development of recombinant thrombin eliminated contamination of bovine FV, and infrequent use of bovine thrombin has decreased the risk of FV inhibitor development. Here, we report the case of a 43-year-old man who had marked prolongation of prothrombin time and activated partial thromboplastin time after surgery. Mixing coagulation studies with normal plasma and patient’s plasma suggested the presence of an inhibitor. Clotting factor assays revealed that FV activity decreased to <1% with positive FV inhibitor titer (9.2 Bethesda units). The diagnosis of the FV inhibitor was confirmed. Overt bleeding was not observed during the course of hospitalization. His coagulation abnormalities rapidly normalized without any medical intervention. A careful review of his medical records revealed that no tissue adhesives were used in the patient, and the FV inhibitor would likely be autoantibodies. Antibiotic use during the perioperative period or the surgical procedure itself may trigger the occurrence of FV inhibitors. This case highlights that FV inhibitor may develop after the surgical procedure even without a history of the use of fibrin tissue adhesives. Surgeons and hematologists should be aware that this rare but potentially life-threatening condition may occur after the surgical procedure.

## Introduction

Development of acquired coagulation factor inhibitors is a rare postoperative complication. Inhibitors develop mostly against factor VIII (FVIII) and von Willebrand factor, while rarely against factors V (FV), XI, XII, and XIII, as well as vitamin K-dependent coagulation factors II (prothrombin), VII (FVII), IX (FIX), and X (FX) [1]. FV inhibitors are known to appear after the surgical procedure. Heteroantibodies developed against bovine FV, a contaminant in bovine thrombin preparations used as surgical fibrin tissue adhesives, cross-react and neutralize human FV [2]. However, as the development of recombinant thrombin eliminated the contamination of bovine FV, the FV inhibitor associated with tissue adhesives has been infrequently reported. Here, we report a case of an FV inhibitor that developed after surgery in a male patient who had marked prolongation in coagulation time, although no tissue adhesives had been used.

## Case presentation

A 43-year-old man presented to our hospital with a complaint of abdominal pain. The patient was referred to our hospital because of obstructive jaundice that occurred two years and ten months ago. After careful examination, the patient was diagnosed with obstructive jaundice due to a cystic mass in the head of the pancreas along with chronic alcoholic pancreatitis which was treated with an oral proton pump inhibitor. However, as the patient was repeatedly hospitalized due to exacerbation of pancreatitis, pancreatoduodenectomy with gastrojejunal anastomosis was performed two years and two months ago. Histopathological examination revealed a cystic lesion without malignancy. However, perforation of the gastrojejunal ulcer occurred twice. Each time, omental patch closure of the perforation was performed. As the patient did not visit our office as scheduled, proton pump inhibitor treatment was frequently interrupted.

Therefore, recurrent perforation was strongly suspected this time. Because all beds in our hospital were full, the patient was referred to another hospital where he underwent urgent patch closure of the perforation and enterostomy. Histopathological examination of the incision site revealed inflammation and necrosis without malignancy. As the clinical condition after the surgery was not significant, the patient was transferred to our hospital on postoperative day (POD) 3. The physical findings were unremarkable without purpura, petechiae, mucosal bleeding, or oozing. Laboratory findings showed elevated D-dimer levels (8.4 mg/L) and within the normal range of prothrombin time (PT)-international normalized ratio (INR) and activated partial thromboplastin time (APTT) (0.91 and 34.2 seconds, respectively). However, on POD 10, hemostatic tests revealed marked prolongation of PT (PT-INR 7.18) and APTT (>150 seconds), although the D-dimer level decreased to 1.8 mg/L. The fibrinogen level (285 mg/dL) was within the normal range. His blood biochemistry was unremarkable. As an underlying cause of abnormal coagulation test results, vitamin K deficiency after fasting and the use of antibiotics was initially suspected. However, vitamin K supplementation failed to shorten PT and APTT. Protein induced by vitamin K absence or antagonist (PIVKA)-II was within the normal range (10 mAU/mL). Taken together, vitamin K deficiency was not observed.

**Figure 1:**
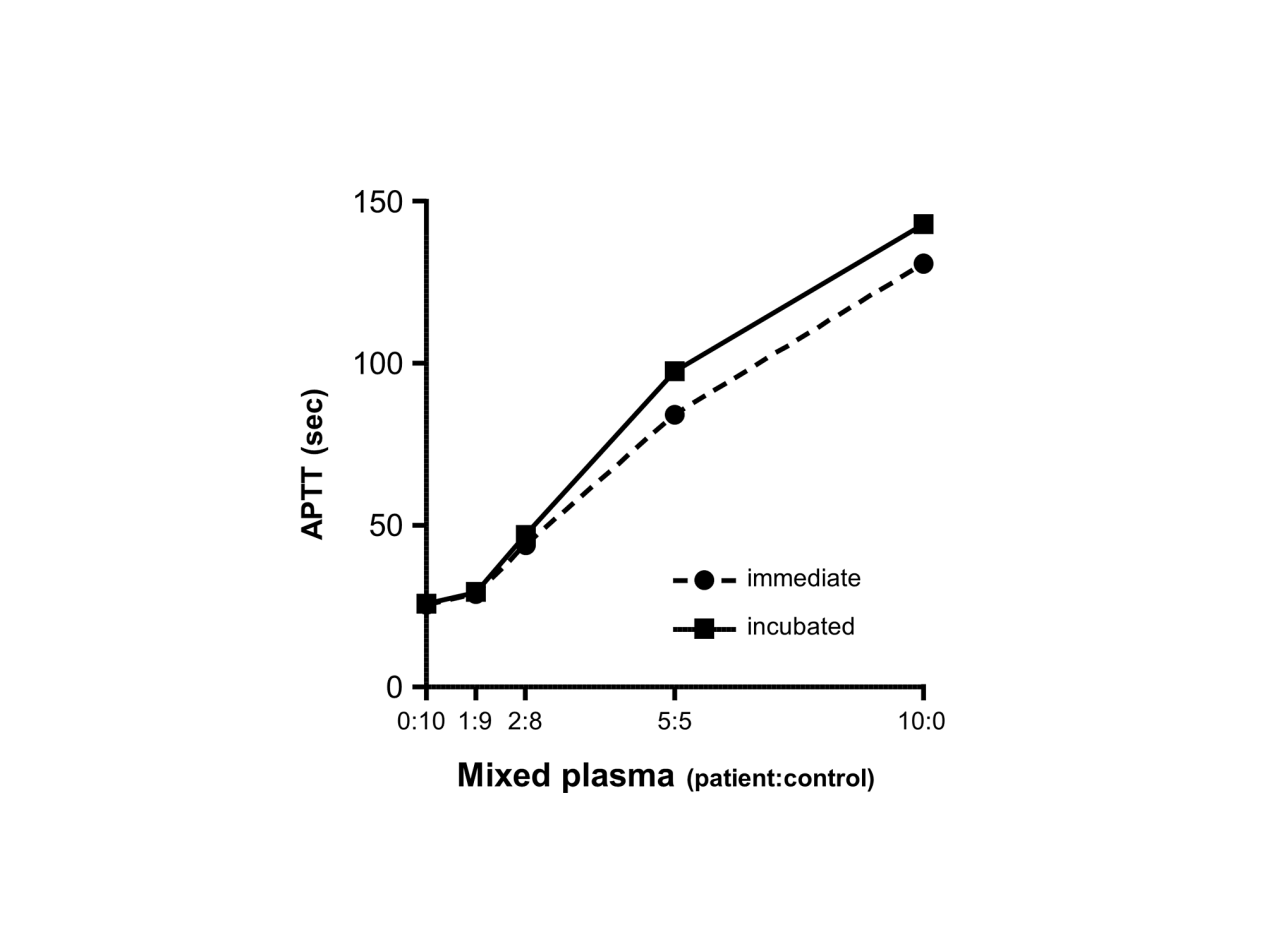
Cross-mixing test. APTT was measured using a mixture of the patient’s plasma and the normal plasma prepared at various ratios with and without incubation for two hours at 37 ˚C. The obtained curve showed an inhibitor pattern. APTT: activated partial thromboplastin time

On POD 18, a hematologist was consulted. Based on the clinical course and laboratory findings, anti-phospholipid syndrome (APS) or acquired coagulation factor inhibitors were suspected. The APTT cross-mixing test revealed a convex upward curve (Figure 1), suggesting the presence of inhibitors, including lupus anticoagulants or coagulation factor-neutralizing antibodies [3]. Blood examination (Table 1) revealed that the anti-cardiolipin-beta-2-glycoprotein I complex antibody (aCL/beta(2)GPI) was negative, while the phospholipid neutralization test was not measurable because clotting did not occur. Furthermore, clinical symptoms were not consistent with those of the APS. Therefore, the APS was unlikely. The cross-mixing test after two-hour incubation at 37 °C showed further prolongation in APTT (Figure 1), indicating coagulation factor inhibitor. Although each factor activity was decreased, FV activity was exceptionally decreased to <1.0%. The thrombotest was almost within the normal range. The FV inhibitor was 9.2 Bethesda units (BU). These results allowed us to diagnose the FV inhibitor. Because there was no bleeding tendency, the patient was observed without any immunosuppressant or platelet transfusion.

On POD 31, both PT and APTT were reduced (PT-INR 3.79 and 77.1 seconds, respectively), and the patient was discharged. On POD 45, both PT and APTT were within the normal range (PT-INR 1.01 and 28.0 seconds, respectively). FV activity was restored to 90%. Spontaneous remission was determined.

**Table 1:** Laboratory findings on POD 18 aCL: Anti-cardiolipin-beta-2-glycoprotein I complex antibody; ALT: alanine aminotransferase; AST: aspartate aminotransferase; CBC: complete blood count; CH50: 50% hemolytic complement activity; CRP: C-reactive protein; Ig: immunoglobulin; n.d.: not determined; POD: postoperative day; TSH: Thyroid-stimulating hormone

CBC		Immune serum	
Red blood cells	3.05×10^12^/L	IgA	141.0 mg/dL
Hemoglobin	10.2 g/dL	IgG	717.0 mg/dL
White blood cels	4.2×10^9^/L	IgM	64 mg/dL
Platelets	398×10^9^/L	IgG4	17.0 mg/dL
Biochemistry		C3	69 mg/dL
CRP	0.1 mg/dL	C4	20.4 mg/dL
Total protein	5.1 g/dL	CH50	36 U/mL
Amylase	22 IU/L	Anti-nuclear antibody	<1:40
AST	24 IU/L	Anti-DNA antibody	<1.7 IU/mL
ALT	7 IU/L	Rheumatoid factor	5 IU/mL
Lactate dehydrogenase	122 IU/L	Lupus anticoagulant	n.d.
Alkaline phosphatase	209 IU/L	aCL	<1.3
Blood urea nitrogen	6.1 mg/dL	Coagulation	
Creatinine	0.53 mg/dL	Factor II (prothrombin)	17.3%
Blood glucose	95 mg/dL	Factor V	<1.0%
Na	133 mEq/L	Factor VII	48.8%
K	3.8 mEq/L	Factor VIII	55.7%
Cl	99 mEq/L	Factor IX	19.8%
Ca	10 mEq/L	Factor X	17.9%
Endocrine		Factor XI	62.8%
TSH	3.864 µU/mL	Factor XII	41.7%
Free triiodothyronine	3.61 pg/mL	Cogulation factors	
Free thyroxine	1.30 ng/dL	Factor II (prothrombin)	17.3%
		Factor V	<1.0%
		Factor VII	48.8%
		Factor VIII	55.7%
		Factor IX	19.8%
		Factor X	17.9%
		Factor XI	62.8%
		Factor XII	41.7%

Furthermore, clinical symptoms were not consistent with those of the APS. Therefore, the APS was unlikely. The cross-mixing test after two-hour incubation at 37 °C showed further prolongation in APTT (Figure 1), indicating coagulation factor inhibitor.

Although each factor activity was decreased, FV activity was exceptionally decreased to <1.0%. The thrombotest was almost within the normal range. The FV inhibitor was proven to be 9.2 BU. These results allowed us to diagnose the FV inhibitor. Because there was no bleeding tendency, the patient was observed without any immunosuppressant or platelet transfusion.

On POD 31, both PT and APTT were reduced (PT-INR 3.79 and 77.1 seconds, respectively), and the patient was discharged. On POD 45, both PT and APTT were within the normal range (PT-INR 1.01 and 28.0 seconds, respectively). FV activity was restored to 90%. Spontaneous remission was determined.

## Discussion

In this case, marked coagulation test abnormalities including prolongation of both PT and APTT were observed. The primary mechanism underlying the prolongation of both PT and APTT is the decreased activity of coagulation factors in the common pathway of the coagulation cascade, that is, fibrinogen, prothrombin, FV, and FX. This may be caused by decreased production or increased consumption of these factors. Because these factors are synthesized in a vitamin K-dependent manner in the liver, conditions such as hepatic insufficiency, vitamin K deficiency, and excessive doses of anti-vitamin K anticoagulant warfarin may inhibit the production. However, disseminated intravascular coagulation or thrombotic microangiopathy in which these factors are aggressively consumed may also decrease these factors. In addition, synthetic anti-thrombin agents such as argatroban, direct oral anticoagulants, and excessive heparin may prolong clotting time. However, in this patient who had no thrombotic tendency, blood biochemistry tests revealed normal liver function and no PIVKA-II elevation. Furthermore, no anticoagulant was used. Therefore, APS or a coagulation factor inhibitor was suspected.

For the diagnosis of APS, both clinical manifestations and laboratory findings are critical. Clinical manifestations include female infertility and thrombosis, while laboratory findings include positive anticardiolipin antibody or lupus anticoagulant [4]. In this case, clinical symptoms did not accord with those of APS. Furthermore, aCL/beta(2)GPI was not detected; hence, APS was excluded.

Acquired coagulation factor inhibitor is a rare and life-threatening condition, which may be noticed incidentally through the prolongation of PT and/or APTT, as seen in this case. Thrombotest, which was established over 60 years ago [5], reflects only three vitamin K-dependent coagulation factors, namely, prothrombin, FVII, and FX, and is not influenced by fibrinogen and FV. As thrombotest time shows normal values for FV inhibitors, the thrombotest is conceived to be useful for the differential diagnosis of FV inhibitor [6]. This case further supports its usefulness in clinical settings. Furthermore, a convex upward curve in the APTT cross-mixing test, prolongation of APTT after incubation, marked decreased activity of FV (<1%), and positive FV inhibitor titer (9.2 BU) led to the definitive diagnosis of FV inhibitor. In a recent systematic review, the median inhibitor titer of acquired FV inhibitor was 9 BU, and the median activity of FV was 2% [7].

Notably, each coagulation factor activity other than that of FV showed low levels in this case. In coagulation factor assays, diluted plasma of a patient mixed with deficiency plasma of the factor of interest is run in a clotting assay (PT or APTT). As FV is essential to converting prothrombin to thrombin, when significant FV-inhibitory activity remains in diluted patient’s plasma, it leads to prolongation of clotting time in any coagulation factor assay but not so much as that in FV assay. To avoid this, chromogenic substrate assays are useful. However, chromogenic substrate assays are available for only some factors. Further, because prolonged clotting time was normalized in a short time, we did not perform the assay.

Inhibitors are classified into three types: spontaneous autoantibodies, alloantibodies in congenital FV deficiency, and cross-reacting anti-bovine FV heteroantibodies [8]. Spontaneous autoantibodies or cross-reacting anti-bovine FV antibodies were possible in this case. In postoperative FV inhibitors, the latter has been reported to be induced by surgical fibrin tissue adhesive comprising thrombin and fibrinogen [9]. Because the thrombin used in tissue adhesives was formerly bovine-derived [10], thrombin preparation was contaminated with a small quantity of bovine FV. Antibodies elicited against heterologous FV might cross-react and neutralize human FV [11]. However, as recombinant thrombin is widely used, bovine FV is eliminated in the current tissue adhesives. The patient in the present case had undergone surgical procedures four times, including this time. A careful review of medical records revealed no use of tissue adhesives in any surgical procedure. Therefore, inhibitors would be spontaneous autoantibodies.

Spontaneous autoantibodies are elicited by an autoimmune mechanism. The triggers of autoimmune diseases include environmental factors that activate immune responses such as infection, stress, drug, injury, surgery, pregnancy, childbirth, and, indirectly, genetic factors by which the immune system easily goes out of control [12-14]. Among them, drugs, surgical procedures, and infections are listed in descending order as the triggers of FV inhibitors [7,15]. A wide range of antimicrobial agents, such as beta-lactams, aminoglycosides, and quinolones, could trigger the development of FV inhibitors [15]. In this case, cefmetazole and meropenem were used in the previous hospital and ours’, respectively. Both antibiotics are suspected drugs. However, because antimicrobial agents are frequently used in surgical procedures and infectious diseases, it will be difficult to establish whether the drug is primarily involved in the development of FV inhibitor. The surgery itself can induce an autoimmune response as with autoimmune Guillain-Barré syndrome and systemic lupus erythematosus [16].

The severity of FV inhibitors differs by case. Although its hemorrhagic manifestation is milder than that in FVIII inhibitor, life-threatening bleeding may occur [11]. On the contrary, the FV inhibitor might cause thrombosis depending on the epitope recognized by the autoantibody [8]. Nonetheless, some are asymptomatic, as in this case. While FV generated in the liver is released into the bloodstream, the FV contained in platelet alpha granules is as much as 20% of that in circulating blood, exhibits resistance to inactivation [17], and is likely to be less affected by inhibitors [18]. This patient did not present with bleeding tendency, probably due to these mechanisms.

Considering the rarity of the condition and the possible risk of life-threatening hemorrhage, involving a hematologist early in the workup would be preferable for timely diagnosis and adequate treatment. While asymptomatic patients usually do not need treatment as in this case, medical intervention is required for patients with a bleeding tendency. As hemostatic therapy for acute hemorrhage, transfusion of platelet concentrates will be the first line. Fresh frozen plasma, prothrombin complex concentrates, or recombinant activated factor VII has also been used [15,19]. Plasma exchange therapy will be effective for the transient removal of inhibitors. The efficacy of immunosuppressants such as corticosteroids and cyclophosphamide and the chimeric anti-CD20 monoclonal antibody rituximab for eradicating inhibitors has been reported [15,20].

## Conclusions

Our experience suggests that surgeons and hematologists should be aware that an FV inhibitor might occur even in patients who do not have tissue adhesives. Fortunately, in our case, no coagulation disorder was observed. The coagulation test abnormality promptly disappeared in the natural course. However, as fatal hemorrhage may occur when coagulation test abnormality is observed, we should conduct further examinations, including cross-mixing tests with coagulation factor inhibitors in mind, and provide an early diagnosis.
